# Giant intrascrotal embryonal rhabdomyosarcoma in an adult: a case report and review of the literature

**DOI:** 10.1186/s13256-018-1607-1

**Published:** 2018-05-28

**Authors:** Wentao Gong, Qingqiang Gao, Zhipeng Xu, Yutian Dai

**Affiliations:** 1 Medical School of Nanjing University, Nanjing, 210093 China; 2 Department of Andrology, Nanjing Drum Tower Hospital, The Affiliated Hospital of Nanjing University Medical School, Nanjing, 210008 China

**Keywords:** Intrascrotal, Embryonal rhabdomyosarcoma, Adult

## Abstract

**Background:**

Intrascrotal embryonal rhabdomyosarcoma in adults is a rare tumor with high aggression and a poor prognosis. We report our patient’s case and review the relevant literature to improve the understanding of this rare disease.

**Case presentation:**

A 21-year-old Han Chinese man presented to our hospital with a right intrascrotal mass of 1 year’s duration. His physical examination revealed an enlarged right scrotum containing a huge tender mass measuring about 10 × 7 cm. Ordinary and contrast-enhanced ultrasonography showed a solid mass in the right scrotum, which was suspected to be a malignant tumor. An abdominopelvic computed tomographic scan revealed metastases in the retroperitoneal lymph nodes. The patient was diagnosed with malignant testicular tumor and underwent a right radical orchiectomy by an inguinal approach. Postoperative pathological examination suggested an intrascrotal embryonal rhabdomyosarcoma.

**Conclusions:**

Intrascrotal embryonal rhabdomyosarcoma is a rare but highly aggressive tumor. Clinical and imaging manifestations of this tumor are nonspecific, so the definitive diagnosis depends on postoperative pathology and immunohistochemistry. Early suspicion, radical orchiectomy, accurate pathologic diagnosis, and adjuvant chemotherapy and/or radiotherapy are the keys to optimal prognosis.

## Background

Rhabdomyosarcoma (RMS) is the most common soft tissue tumor in children, but it is rare in adults [1, 2]. Intrascrotal tumors originate primarily from germ cells, whereas non-germinal cell tumors are uncommon [3]. An adult patient admitted to our hospital had a giant intrascrotal embryonal RMS. This report describes the pathogenesis, clinical manifestations, diagnosis, treatment methods, and prognosis of intrascrotal embryonal RMS in our patient’s case and in other cases previously reported in the literature, with the aim of improving the understanding of this rare disease.

## Case presentation

Our patient was a 21-year-old Han Chinese man who had found a painless testicular mass in his right scrotum 1 year before presentation to our hospital, for which he had gone to another hospital for treatment. He received no definite diagnosis there but was given a 1-week Chinese herb decoction. Owing to the loss of the previous case record, the suspected diagnosis and the names of the herbs were unknown. He observed no obvious improvement. The mass remained small and unchanged during the first 9 months of the disease course and therefore did not arouse the patient’s attention enough to seek further treatment. However, over the next 3 months, the mass enlarged rapidly and led to obvious right scrotal tenderness. Ultrasonography showed a solid space-occupying lesion measuring 9.7 × 7.7 cm in the right scrotum. The patient reported no obvious fever, osphyalgia, abdominal pain, frequent micturition, urgency, dysuria, or gross hematuria.

The patient was admitted to our andrology department in October 2017. On admission, we took a full medical history, including his personal and family history as well as previous treatment of his testicular mass. He had no history of hypertension, hyperlipidemia, coronary heart disease, type 2 diabetes mellitus, traumas, or surgeries, among others. He did not smoke tobacco or consume alcohol, and he had no family history of testicular tumor.

The patient’s physical examination revealed unremarkable vital signs (body temperature 37.1 °C, heart rate 70 beats/minute, blood pressure 120/65 mmHg, respiration 17 breaths/minute), normal heart and lung sounds, and a soft abdomen with no tenderness or organomegaly. Urogenital palpation disclosed an enlarged right scrotum with a hard, tender mass (~ 10 × 8 cm) adhering to the right testis and epididymis, but no palpable masses in the right spermatic cord or bilateral inguinal regions. The patient’s neurological examination showed no abnormalities.

**Fig. 1:**
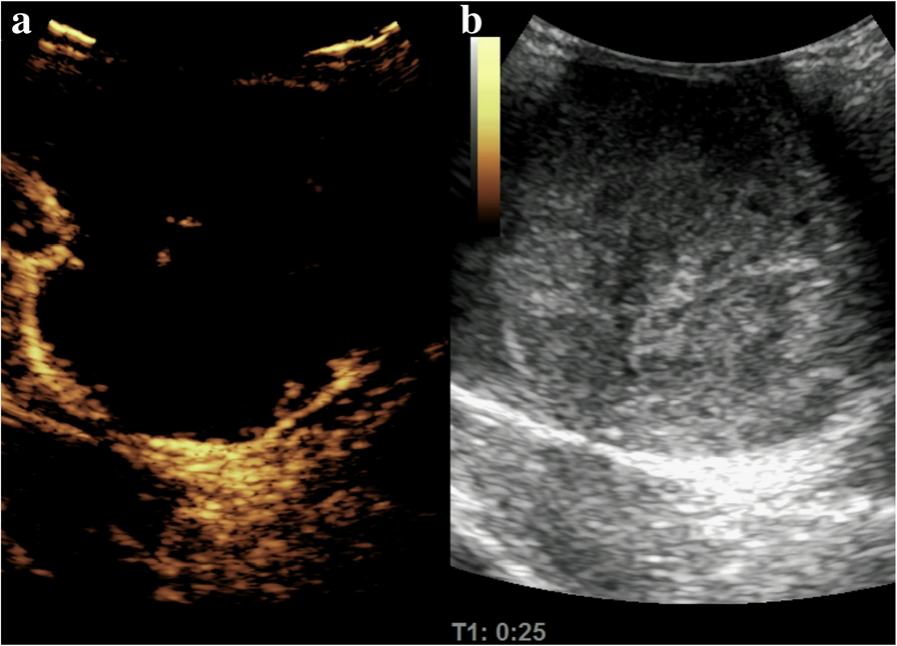
**a** Contrast-enhanced Ultrasonographic images showing that the mass enhanced from the periphery at 25 seconds after the bolus injection of SonoVue contrast agent. The coarse nourishing blood vessels are clearly displayed. **b** The corresponding two-dimensional ultrasound shows the morphologically abnormal right testis with heterogeneous internal echoes

**Fig. 2:**
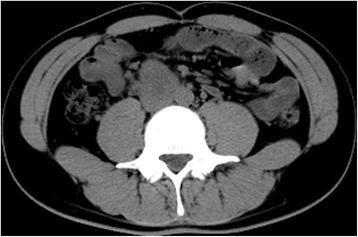
Computed tomographic scan displaying the suspicious metastasis sites in the retroperitoneal lymph nodes (the soft tissue density shadows anterior to the right psoas and at the level of the fourth lumbar vertebra)

Color Doppler ultrasonography displayed a solid intrascrotal mass (11.5 × 8.2 × 7.6 cm) with heterogeneous inner echoes and short linear blood vessel flow signals at the mass periphery. Contrast-enhanced ultrasonography (CEUS) (SonoVue contrast agent; Bracco Diagnostics, Monroe Township, NJ, USA) showed a partial enhancement that appeared mainly in the periphery of the intrascrotal lesion during arterial phase; the lesion also showed an extensive interior filling defect. The enhanced part was irregular in form and contained coarse, twisted blood vessels (Fig. 1a-b). The CEUS results suggested a testicular germ cell tumor. Abdominopelvic computed tomography (CT) revealed some soft tissue density shadows anterior to the right psoas (and at the level of the fourth lumbar vertebra), which were suspected to be metastases in the retroperitoneal lymph nodes (RPLNs) (Fig. 2). Both the physical examination and the imaging results showed no abnormalities of the left testis, epididymis, or spermatic cord. No significant abnormal signs were found on chest x-ray film.

The results of the patient’s complete blood count, blood biochemistry, and urinalysis were all normal, except for a high lactate dehydrogenase concentration (295 U/L). His α-fetoprotein (AFP; < 1.3 ng/ml) and human chorionic gonadotropin (HCG; < 0.1 mIU/ml) concentrations were within normal ranges.

All these findings, taken together, indicated that the right intrascrotal mass was a malignant tumor. The patient rejected retroperitoneal lymph node dissection (RPLND), but he underwent a right radical orchiectomy by inguinal approach. Intraoperative pathology suggested a small cell carcinoma of the right testis.

**Fig. 3:**
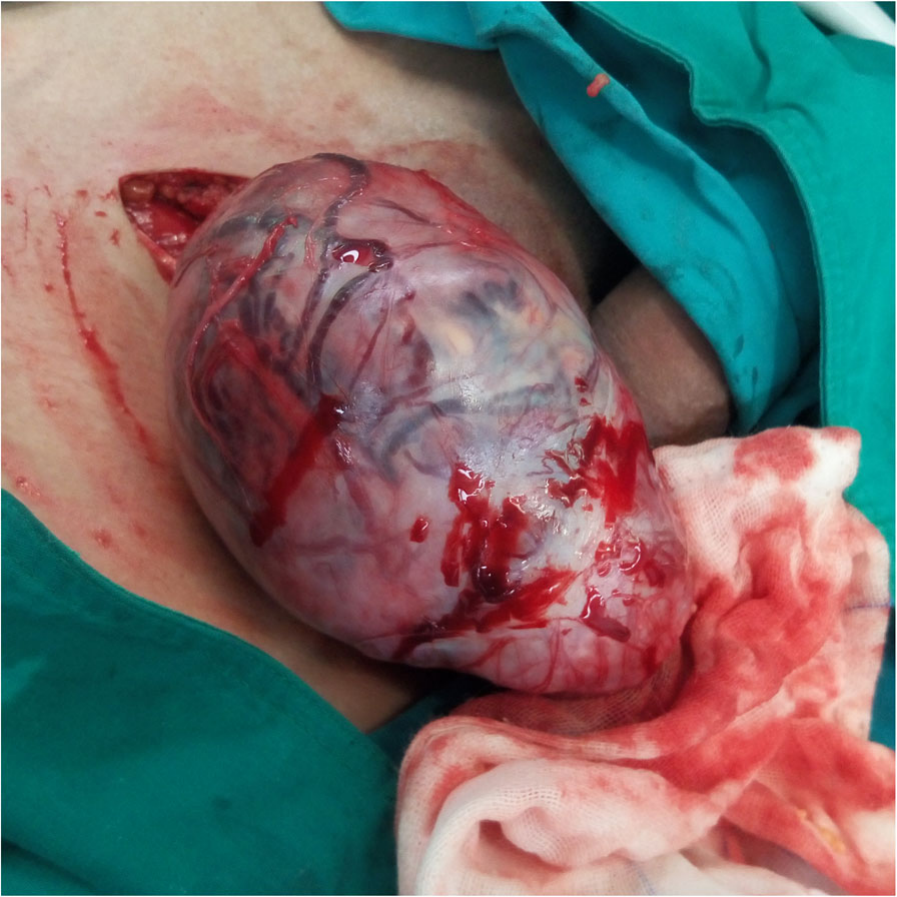
Gross specimen of the giant right intrascrotal tumor measuring 10 × 7 × 6 cm

**Fig. 4:**
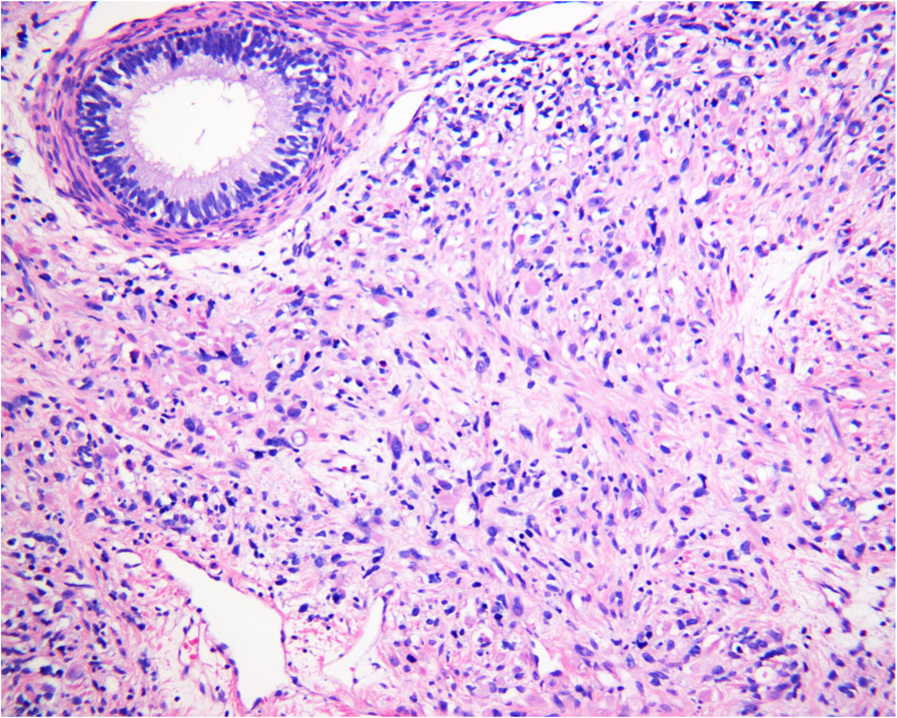
Postoperative pathologic section (H&E stain, original magnification × 200) showing diffuse distribution of small round cells with obvious atypia

**Fig. 5:**
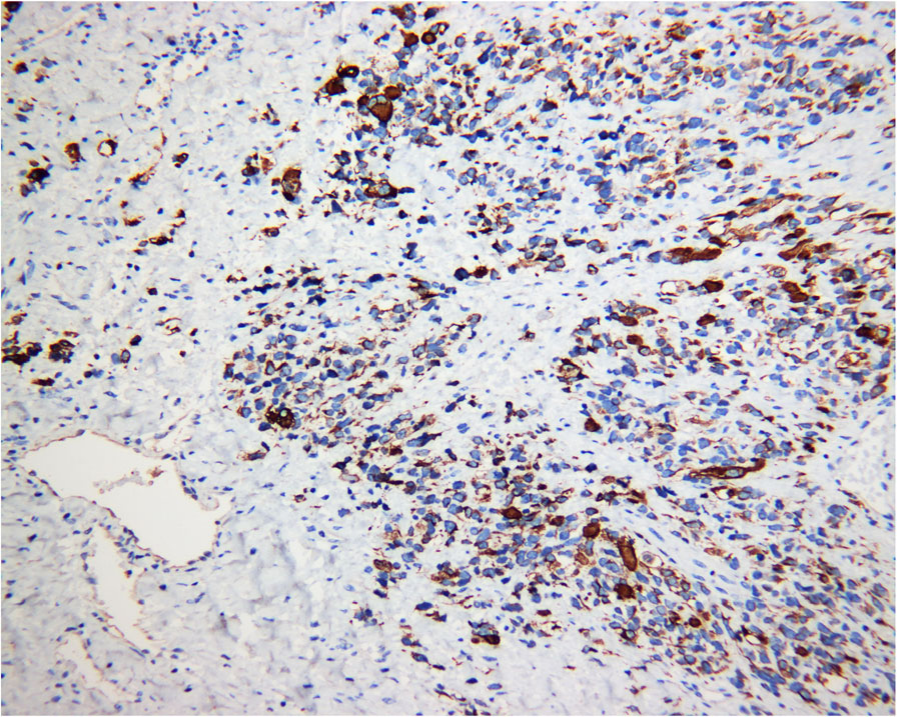
A specific immunohistochemical result of rhabdomyosarcoma: desmin-positive tumor cells (immunohistochemical stain, original magnification × 200)

Postoperative pathology showed a giant (10 × 7 × 6 cm) intrascrotal tumor that involved the right testis, epididymis, and paratesticular tissues (Fig. 3). Microscopy showed diffuse distribution of small round cells with obvious atypia (Fig. 4). Tumor emboli were found in the surrounding vessels. No nerve was infiltrated by the tumor tissue. The incisal edge of the right spermatic cord was negative. Immunohistochemistry showed the tumor tissue to be negative for cytokeratin, calretinin, inhibin-α, placental alkaline phosphatase, lymphocyte common antigen, S100, anaplastic lymphoma kinase, α-smooth muscle actin, CD34, and SOX10. Positive immunohistochemistry results were found for vimentin (+), CD56 (+++), myogenin (+++), myoblast determination protein 1 (MyoD1) (++), desmin (+++), and Ki-67 (70%+) (Fig. 5). The histopathologic diagnosis was embryonal RMS.

According to all the clinical, imaging, and pathologic evidence, the tumor was finally diagnosed as an intrascrotal embryonal RMS. Its exact origin was hard to confirm, owing to its extensive invasion of testis, epididymis, and paratesticular tissues. Because the patient rejected RPLND, we could not verify whether the tumor had metastasized to the RPLNs. The patient is now undergoing vincristine, actinomycin D, and cyclophosphamide (VAC) chemotherapy regimen, combined with abdominopelvic radiotherapy, at another hospital, which was initiated 20 days after discharge from our department. We will continue to follow-up of our patient.

## Discussion

We present the medical history, diagnostic procedure, and treatment of an adult patient with intrascrotal embryonal RMS. Because few studies of intrascrotal embryonal RMS in adults are available, we hope that this case report and the following literature review will contribute to the understanding, diagnosis, and treatment of this rare disease.

RMS is one of the most common pediatric tumors, comprising up to half of all soft tissue sarcomas [1, 2]. However, adult RMS is relatively rare, accounting for only 3% of all soft tissue sarcomas [1, 2]. In addition, intrascrotal tumors originate primarily from germ cells; non-germinal cell tumors are uncommon [3]. According to the epidemiological characteristics of RMS and intrascrotal tumors, adult intrascrotal RMS is particularly rare. Intrascrotal RMS can originate from testis or from paratesticular tissues. Perhaps primary testicular RMS arises from undifferentiated mesenchyme that retains the capacity for rhabdomyoblastic differentiation or from embryonal muscle tissue that has been misplaced at the early stages of tissue development [3]. Paratesticular RMS is thought to arise from the mesenchymal elements of the epididymis or spermatic cord [4].

The typical clinical presentation of intrascrotal embryonal RMS is a painless unilateral enlargement of the scrotum, usually over the course of a few weeks [3]. Bilateral groins should be palpated to assess if the tumor has metastasized to inguinal lymph nodes. Ordinary ultrasonography and CEUS can help to differentiate between benign and malignant intrascrotal tumors and to evaluate the tumor’s extent of infiltration [5, 6]. CT is often used to look for RPLN metastases [7]. However, all the clinical presentations and imaging manifestations of intrascrotal embryonal RMS are not specific. Furthermore, serum biomarkers such as β-HCG and AFP are also nonspecific for this kind of tumor [8]. The limitations of traditional diagnostic methods make accurate presurgical diagnosis of intrascrotal embryonal RMS difficult, and definitive diagnoses therefore depend on postoperative pathologic examination. Observation of the gross specimen is the key to confirming the origin of the tumor [9]. In our patient, however, the tumor extensively involved the testis, epididymis, and paratesticular tissues, so we could not determine its exact origin. Microscopic images of embryonal RMS are characterized by diffuse distribution of small round cells with obvious atypia [10]. However, intraoperative pathology suggested that the tumor in our patient was a testicular small cell carcinoma. This indicates that embryonal RMS should be differentiated from other tumors that are rich with small round cells, such as small cell carcinoma, neuroblastoma, or lymphoma.

Immunohistochemical staining is now the optimal diagnostic method for RMS [11], which is typically positive for one or more muscle-specific markers, including desmin, muscle-specific actin, MyoD1, myoglobin, and/or myogenin [4, 12, 13]. In our patient, the tumor was positive for desmin, myogenin, and MyoD1. On the basis of these results and the microscopic images, the tumor was diagnosed as an intrascrotal embryonal RMS.

Because RMS rarely occurs in adults, treatments used in pediatric patients are often applied to adult cases. The standard treatment for intrascrotal embryonal RMS is radical orchiectomy combined with adjuvant chemotherapy and radiotherapy [14]. The Intergroup Rhabdomyosarcoma Study Group (IRSG) classifies embryonal RMS into four groups by their pathologic margins and lymph node metastasis status, and it recommends respective targeted treatments [7]. Therefore, RPLND is of great importance in guiding postoperative therapy because it can confirm the appropriate IRSG group. Nerve preservation RPLND is generally recommended when the imaging findings suggest RPLN metastases [15]. However, whether imaging-negative patients need RPLND is still a controversial problem. Walterhouse and Watson administered chemotherapy alone to imaging-negative patients who had undergone radical orchiectomy; only one patient (16.7%) had a regional recurrence during the follow-up period, and that patient was saved with additional therapy [14]. This result indicates that imaging-negative cases can avoid RPLND, thus reducing complications and improving quality of life. Contrarily, Wiener *et al*. found that CT scans for patients with intrascrotal RMS (especially for those over 10 years old) often underestimate RPLN metastases from localized tumors, so they urged that imaging-negative adolescent patients should still undergo RPLND for further assessment of nodal involvement and to provide guidance for subsequent therapies [16].

Postoperative chemotherapy and/or radiotherapy can dramatically increase the survival rate of pediatric patients with RMS [17]. Adjuvant chemotherapy is now recommended as a standard therapy for patients with RMS in all IRSG groups [18]. Commonly used chemotherapeutic agents for RMS include VAC [4]. Specific chemotherapy regimens should be chosen according to IRSG classification [12]. Moreover, chemotherapy can downgrade unresectable tumors and create opportunities for surgical treatment [4]. However, the effect of chemotherapy for adult patients is still controversial [2]. Hawkins *et al*. suggested that chemotherapy cannot provide a significant survival benefit to patients over 21 years old [19], whereas Ferrari *et al*. retrospectively analyzed 171 adult patients with RMS and found that their rate of response to chemotherapy was 85% [20]. Although this rate was lower than that for children, it was significantly higher than for other kinds of adult sarcomas and indicated that chemotherapy is effective for adult patients with embryonal RMS. Radiotherapy for residual lesions or regional lymph nodes is recommended only for patients with microscopic or macroscopic remnant tumor tissue and/or metastasis sites; it cannot bring obvious benefit to patients with IRSG group I disease [21]. Although it is generally recognized that the experiences originating from the treatment of pediatric patients could bring benefits to adult cases, there are still no evidence-based chemotherapy or radiotherapy strategies for adult patients in each IRSG group, owing to the rarity of intrascrotal embryonal RMS in the adult population. As for follow-up strategies, patients with RMS should be monitored for tumor recurrence or metastasis and adverse effects related to operations, chemotherapy, and/or radiotherapy just as for patients with other kinds of cancers [14].

Intrascrotal RMS has a poor prognosis [3]. The 1-year overall survival (OS) rate is 68%, and the 5-year OS rate is 30% [3]. RPLN metastasis is an important prognostic factor [17]. Ferrari *et al*. found that the 5-year disease-free survival was 97% for patients without RPLN metastases and 42% for those with metastasis [17]. Another prognostic factor is the age of the patient [4]. Compared with pediatric patients with RMS, adults with RMS of any organ have significantly worse long-term outcomes [4].

In our patient, CT findings suggested RPLN metastases, but because the patient rejected RPLND, we could not confirm the IRSG classification of his embryonal RMS. If the soft tissue density shadows seen by CT were metastases, the tumor would be classified as IRSG group IIA. In view of the aggressiveness and poor prognosis of adult RMS, our patient should be treated according to the therapeutic regimen proposed by the IRSG for group IIA disease. In fact, the patient is continuing to receive a VAC chemotherapy regimen with abdominopelvic radiotherapy in another hospital. A longer follow-up for him has been planned.

## Conclusions

Intrascrotal embryonal RMS is a rare but aggressive tumor, especially in adults, and therefore warrants careful attention for accurate diagnosis and appropriate treatment. Although the definitive diagnosis of embryonal RMS depends on postoperative pathology, physical examination and imaging tests can establish clinical suspicion and detect metastases. Current treatment for adult patients is based mostly on treatment for children. Early suspicion and radical orchiectomy are especially important to achieving an optimal prognosis. Adjuvant chemotherapy or radiotherapy can prolong survival and elevate the survival rate.

## Abbreviations

AFP: α-Fetoprotein
CEUS: Contrast-enhanced ultrasonography
CT: Computed tomography
HCG: Human chorionic gonadotropin
IRSG: Intergroup Rhabdomyosarcoma Study Group
MyoD1: myoblast determination protein 1
OS: Overall survival
RMS: Rhabdomyosarcoma
RPLN: Retroperitoneal lymph node
RPLND: Retroperitoneal lymph node dissection
VAC: Vincristine, actinomycin D, and cyclophosphamide chemotherapy
